# Working nurses’ empathy with patients in public hospitals

**DOI:** 10.1590/1518-8345.6591.3969

**Published:** 2023-08-04

**Authors:** Yolanda Dávila Pontón, Víctor Patricio Díaz Narváez, Bernardo Montero Andrade, Joseline Janeth López Terán, Alejandro Reyes-Reyes, Aracelis Calzadilla-Núñez

**Affiliations:** 1 Universidad del Azuay, Facultad de Medicina, Cuenca, Ecuador.; 2 Universidad Andres Bello, Facultad de Odontología, Santiago, Región Metropolitana, Chile.; 3 Universidad Santo Tomás, Facultad de Ciencias Sociales y Comunicación, Concepción, Región del Bío Bio, Chile.; 4 Universidad Bernardo O’Higgins, Departamento de Investigaciones, Santiago, Región Metropolitana, Chile.

**Keywords:** Cross-Sectional Study, Correlation of Data, Empathy, Nursing Staff, Public Hospital, Ecuador, Estudio Transversal, Correlación de Datos, Empatía, Personal de Enfermería, Hospital Público, Ecuador, Estudo Transversal, Correlação de Dados, Empatia, Recursos Humanos de Enfermagem, Hospital Público, Equador

## Abstract

**Objective:**

to determine the levels of empathy in professional nurses of a high-complexity hospital, to relate age to empathy (and each one of its dimensions), and to establish if there are differences between these levels according to the type of working schedules.

**Method:**

comparative, correlational and cross-sectional design. The sample used (n=271) constituted 40.9% of the total number of nursing professionals. Psychometric properties of the Jefferson Scale of Empathy for Health Professionals were studied. Descriptive statistics were calculated: mean and standard deviation. The association between empathy and age was estimated using regression equations and statistical significance of the regression coefficients, after evaluating the type of curve using variance analysis.

**Results:**

the underlying model of three dimensions of empathy was identified. The values of the descriptive statistics observed were relatively low in empathy and its dimensions. Empathy levels were not associated with the age range. No differences in empathy were found between the types of work schedules. Variability was found in the dimensions: “compassionate care” and “Walking on the patient’s shoes”.

**Conclusion:**

these results show that the levels of empathy observed may imply a deficient performance in empathetic care for patients.

## Abstract (es)

**Objetivo:**

determinar los niveles de empatía en profesionales de enfermería de un hospital de alta complejidad, relacionar la edad con la empatía (y cada una de sus dimensiones), y establecer si existen diferencias entre estos niveles según el tipo de jornada laboral.

**Método:**

diseño comparativo, correlacional y transversal. La muestra utilizada (n=271) constituyó el 40,9% del total de profesionales de enfermería. Se estudiaron las propiedades psicométricas de la Escala de Empatía de Jefferson para Profesionales de la Salud. Se calcularon estadísticos descriptivos: media y desviación estándar. La asociación entre empatía y edad se estimó mediante ecuaciones de regresión y significancia estadística de los coeficientes de regresión, luego de evaluar el tipo de curva mediante análisis de varianza.

**Resultados:**

se identificó el modelo subyacente de las tres dimensiones de la empatía. Los valores de los estadísticos descriptivos observados fueron relativamente bajos en empatía y sus dimensiones. Los niveles de empatía no se asociaron con el rango de edad. No se encontraron diferencias en la empatía entre los tipos de horarios de trabajo. Se encontró variabilidad en las dimensiones: “cuidado compasivo” y “ponerse en los zapatos del paciente”.

**Conclusión:**

estos resultados muestran que los niveles de empatía observados pueden implicar un desempeño deficiente en el cuidado empático de los pacientes.

## Abstract (pt)

**Objetivo:**

determinar os níveis de empatia em enfermeiros profissionais de um hospital de alta complexidade, relacionar a idade com a empatia (e cada uma das suas dimensões) e verificar se existem diferenças entre esses níveis, de acordo com o tipo de horário de trabalho.

**Método:**

delineamento comparativo, correlacional e transversal. A amostra utilizada (n=271) constituiu 40,9% do total de profissionais de enfermagem. Foram estudadas as propriedades psicométricas da Escala de Empatia de Jefferson para Profissionais da Saúde. Foram calculadas estatísticas descritivas: média e desvio padrão. A associação entre empatia e idade foi estimada por meio de equações de regressão e significância estatística dos coeficientes de regressão, após avaliação do tipo de curva por meio de análise de variância.

**Resultados:**

o modelo subjacente de três dimensões de empatia foi identificado. Os valores das estatísticas descritivas observados foram relativamente baixos em empatia e suas dimensões. Níveis de empatia não foram associados com a faixa etária. Não foram encontradas diferenças de empatia entre os tipos de horários de trabalho. Foi encontrada variabilidade nas dimensões: “cuidado compassivo” e “colocar-se no lugar do paciente”.

**Conclusão:**

esses resultados mostram que os níveis de empatia observados podem implicar em um desempenho deficiente no atendimento empático aos pacientes.

**Highlights:**

**(1)** The levels of empathy are low in the nursing professionals studied.

**(2)** These levels are not associated with age and type of work performed.

**(3)** Low levels of empathy could imply a negative alteration of humanized attention.

## Introduction

Assistance activity in the field of health must be permeated by humanization^(^ 1 ^)^. The humanization of nursing professionals in patient care is one of the essential aspects of their work^(^ 2 ^-^ 3 ^)^. Empathy is an attribute that plays an important role in the patient care process and allows an intersubjective connection between two human components of health care: nurses and patients^(^ 4 ^)^. Therefore, empathy is one of the constituent elements of the structure of the concept of humanization in patient care^(^ 2 ^)^.

Empathy is structured by cognitive^(^ 5 ^)^ and emotional^(^ 6 ^)^ factors or components. Cognitive factors are sensitive to teaching-learning processes^(^ 7 ^)^ throughout life, but emotional aspects (especially compassionate care) are complex processes^(^ 8 ^)^ and are difficult to modify^(^ 9 ^)^. The complexity of empathy lies in the fact that cognitive and emotional factors are constantly interacting with each other dialectically^(^ 5 ^-^ 7 ^)^ and, as a consequence, they form a system. Therefore, a “deficit” of any of them (in whatever form) will necessarily imply an alteration of the empathy “system” that, according to the “severity” of such insufficiency (or simply the absence of some of these factors), will determine a decrease in empathy until its cancellation as a complex attribute. This “decrease” of empathy may be due to a “failure” of the capacity and ability to understand or read what the other person thinks and take perspective about this understanding, or compassion for the other person’s physical and/or mental pain^(^ 10 ^)^.

Currently, technological development has allowed the creation of complex instruments that participate in the care of patients. Therefore, the presence of the risk that nurses prioritize technical assistance over the humanized attention of the nursing staff is admissible. Nursing practice requires an understanding of the intimate relationship of nursing (as a profession), the sense of care and being aware of internal ethical values^(^ 11 ^)^ and having prosocial attributes that this profession naturally demands^(^ 12 ^)^.

In professional nursing practice, the core of care must necessarily be present because this core transforms this practice into a real action consisting of the best care for the patient. In addition, the concept of care, it is also necessary for the nursing professional to have a conception that contemplates an integrated understanding of care, suffering, health, the environment, and the person as a human being^(^ 13 ^-^ 17 ^)^. In this sense, empathy is an attribute that makes it possible to mitigate or control all the factors that may have a negative impact on patient care, enhances said care and increases the possibility that the necessary intersubjectivity can be generated so that the nursing professional can perform with high degrees of success^(^ 18 ^-^ 19 ^)^. Therefore, the importance of empathy consists in the practical fact of establishing the appropriate interaction with the patient to provide the humanized care that every patient needs and, at the same time, generate human satisfaction in the patient under care. Empathy is considered as a modulator of the factors that positively or negatively influence this attribute^(^ 9 ^-^ 12 ^)^. The acquisition of empathy is not a purely innate attribute but is formed through complex processes during a person’s natural development^(^ 12 ^-^ 20 ^)^. Due to the complexity of this attribute, the empathic training of future nursing professionals should be the object of attention from the first years in the teaching-learning process^(^ 20 ^-^ 29 ^)^.

There are several instruments to measure empathy, among which are those that have a cognitive approach, for example: Hogan Empathy Scale (EM); affective, for example: Questionnaire Measure of Emotional Empathy (QMEE); and integrators, for example: Interpersonal Reactivity Index (IRI)^(^ 30 ^)^. At present, the most used is the integrative vision^(^ 31 ^)^. However, the measurement of empathy in students and health sciences professionals required an instrument that measured empathy but in a precise context: empathy with the patient. The Jefferson Medical Empathy Scale^(^ 32 ^-^ 33 ^)^, the Jefferson Medical Empathy Scale for medical students (Version-S), emerged. This scale has been adapted for different specialties, including the Empathy Scale for Health Professionals (HP) and the Health Sciences Student Version (HP-S). All these adaptations are characterized by having a good internal consistency: alpha [0.75; 0.89]. Convergent validity has been confirmed by significant correlation coefficients between Jefferson Scale of Empathy (JSE) scores and conceptual measures of compassion. The same occurred with discriminant validity due to the lack of significant association with irrelevant conceptual measures such as self-protection^(^ 31 ^-^ 32 ^)^. Authors^(^ 34 ^)^ have recently published a paper that exhaustively describes other characteristics of this scale, which has been used in its different versions to measure students and health professionals. This scale is also characterized by its stability. Studies that have used this scale report that it repeatedly maintains the three dimensions stable: two cognitive, Perspective Taking (PT) and “Walking in Patient Shoes” (WIPS), and one emotional, Compassionate Care (CC). The facts described above justify the use of the JSE in the study of empathy with the patient in professionals and students of health sciences due to the results observed in the psychometric and trustworthiness studies.

Studies of empathy in professional nurses are scarce, but they provide relevant information that must be studied and explained^(^ 16 ^-^ 19 ^)^, with greater production in populations of nursing students^(^ 9 ^,^ 13 ^-^ 16 ^)^. In the first case, these studies dealt with empathic performance in adverse work conditions and patients’ perception of the attitude of nurses, and, in the second case, they evaluated the levels of empathy in the training process of nursing studies. However, there are few studies on nursing professionals in Latin America that massively evaluate the empathy of nursing staff with the patient and, at the same time, practice in highly complex hospitals in relatively large cities. It is also unknown how the levels of empathy with the patient are distributed in relation to the different types of work schedules (hour load) even though there are studies that establish some degree of relationship between this type of load and the presence of depressing factors, for example, Burnout^(^ 35 ^)^. On the other hand, it has been observed that, in general, the published literature refers that age is not correlated with the levels of empathy in nursing students and professionals and concludes, implicitly or explicitly, that this variable does not seem to be important^(^ 13 ^-^ 16 ^)^. Consequently, the theoretical and practical meaning of the absence of this correlation has not been discussed about the causes that can produce it and the effect that this could have on patient care, especially of those nursing professionals who show low or insufficient levels of empathy.

This paper aims to determine the levels of empathy (and its dimensions) in nursing professionals from a highly complex hospital, relate age to empathy (and each of its dimensions) and establish whether there are differences between them. To meet this objective, it is necessary to previously submit the empathy data to psychometric studies to confirm the structure of three underlying dimensions in the empathy construct in relation to the data observed in the present study^(^ 9 ^,^ 20 ^,^ 26 ^,^ 33 ^-^ 34 ^)^.

## Method

### Design

Comparative, descriptive and cross-sectional study.

### Participants

The sample is composed of 271 professional nurses at a public hospital in Cuenca, Ecuador. This sample corresponds to 40.9% of the total number of nursing professionals working at the mentioned hospital (N=663). The participation of the people evaluated was voluntary. The sampling was convenience. The Hospital where the study was conducted (May 2022) is classified as Third Level (High Complexity). It is run by a decentralized public entity that belongs to the Ecuadorian Social Security Institute (IESS) whose non-delegable purpose is the provision of Compulsory General Insurance throughout the national territory^(^ 36 ^)^.

### Instruments

Empathy was measured using the Jefferson Scale of Empathy version for Health Professionals (JSE-HP version). This scale is a psychometrically sound instrument developed specifically to measure physicians’ empathetic orientation in the context of patient care. It is made up of 20 items and each one of them is evaluated using a Likert scale (from one to seven points with a total of 140 points) and the higher the score, the greater the empathic orientation. It is structured by three factors: Compassionate Care (CC), Perspective Taking (PT), and “Walking in the Patient’s Shoes” (WIPS)^(^ 7 ^,^ 18 ^-^ 20 ^,^ 37 ^)^. The findings support the underlying factor structure of the Jefferson Empathy Scale in a Hispanic-American sample^(^ 38 ^)^.

### Procedure

The translation and adaptation of the JSE-HP were carried out through the process of translation and retro-translation of the original instrument in English^(^ 39 ^)^. Subsequently, it was subjected to a pilot study made up of 30 nursing professionals, drawn from the same study population, to verify the understanding of the questions. Finally, the underlying three-dimensional model was verified by factorial analysis establishing factorial validity.

### Data analysis

Before to data analysis, the Kolmogorov-Smirnov statistic was evaluated to test univariate normality and Mardia’s multivariate kurtosis coefficient^(^ 40 ^)^ to check if the data presented multivariate normality. Subsequently, the various descriptive statistics were calculated, and a confirmatory factor analysis (CFA) model was established based on the Maximum Likelihood method and using Bootstrap, simulating 5000 samples, as a technique that allows making a better fit in the context of the absence of multivariate normality^(^ 41 ^)^. To assess the fit of the CFA model, various goodness-of-fit indices were used: chi-square, the comparative fit index (CFI > .90); Tucker Lewis index (TLI > .90), goodness-of-fit index (GFI > .95) the root mean square error approximation index (RMSEA < .10); and the standardized root mean square residual (SRMR < 0.05), the magnitude of the factor loadings (> .50), and the reliability of the construct with the McDonald’s omega coefficient and Cronbach’s alpha (> .70).

The following descriptive statistics were estimated: arithmetic mean, standard deviation, standard error of the mean, coefficient of variation (CV), confidence interval (CI), and minimum and maximum values of empathy and its dimensions. The association between age (independent variable) and levels of empathy and its dimensions (dependent variables) was made by estimating the regression equation with standardized data and transforming it to a logarithmic scale, analysis of variance (ANOVA) was carried to evaluate the significance of the coefficient of regression and sequential ANOVA to determine the type of curve. The standard deviation of the regression curve and the adjusted and unadjusted variance were estimated. Finally, the comparisons between the levels of empathy and its dimensions between the two types of working schedules were compared using a Mann-Whitney U test, after comparing homoscedasticity using the Levene test. SPSS 25.0, AMOS 25, and Minitab 18.0 programs were used. The level of significance used was α <0.05.

### Ethical aspects

The participation of nursing professionals was voluntary and confidential. The participants signed informed consent before taking the measurements, adjusted to the ethical principles of the Declaration of Helsinki. This study was approved by the Research Ethics Committee of the University of Azuay (CISH-UDA), with a resolution issued on June 17, 2020.

## Results

### Sample characteristics

The sample consisted of 259 women (95.6%) and 12 men (4.4%), aged between 22 and 60 years (Mean=36.59, Standard deviation=8.54, Confidence interval= [35.57; 37.61]). Specifically in men: Mean=31.58, Standard deviation= 7.064; Confidence interval= [27.10; 36.07] and in women: Mean=36.84; Standard Deviation= 8.546; Confidence interval= [35.78; 37.87].

### Assessment of normality

Before data analysis, compliance with the normality assumption was tested. Observe a significant Kolmogorov-Smirnov statistic for all the empathy variables (p<.001), indicative of the absence of univariate normality. With the absence of multivariate normality when observing a multivariate kurtosis coefficient of Mardia^(^ 40 ^)^ of 111.621 (critical ratio= 30.971).

### Confirmatory factor analysis

**Figure 1 -:**
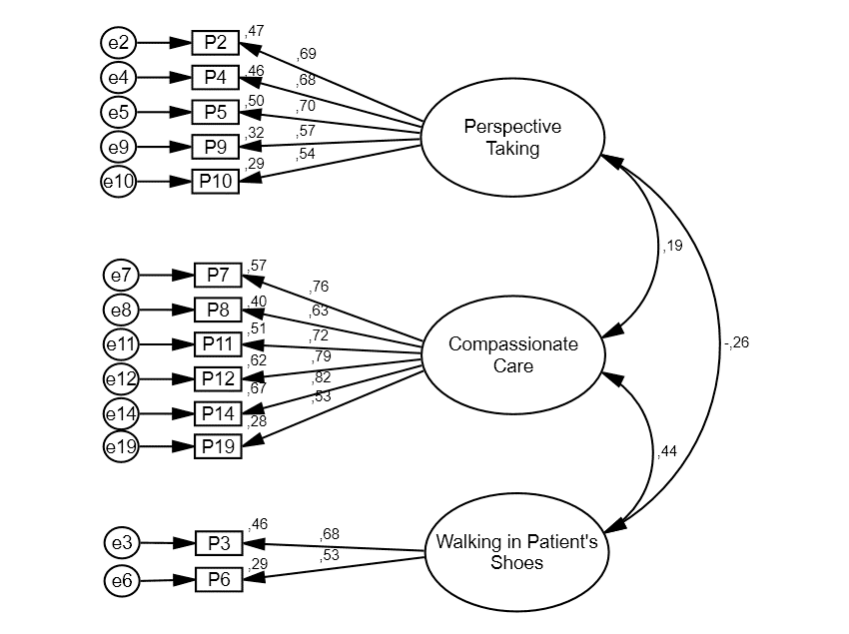
Three-factor model of the Jefferson Empathy Scale in Nurses (JSE-HP version)

To provide evidence of the validity of the empathy construct, confirmatory factor analysis is used, observing a bad adjustment of the data to the three-factor model of empathy proposed by Hojat (2002) \begin{equation*}(\chi^2=379.981, df= 167, p= .0001; \chi^2/df= 2.275, GFI= .876, TLI= .84, CFI = .86, RMSEA=.069 [90\% CI= .060 - .078], SRMR= .070)\end{equation*} , with factorial weights that vary from \begin{equation*}\lambda= .11\end{equation*} to \begin{equation*}\lambda=.82\end{equation*} . Based on the above, it was decided to respecify the model, eliminating items with factor loadings lower than 0.50^(^ 42 ^)^. Retaining the three original factors, but only 16 items, with significant factor loadings that vary between \begin{equation*}\lambda = .53\end{equation*} and \begin{equation*}\lambda= .82\end{equation*} . Adequate goodness-of-fit indices, were observed \begin{equation*}(\chi^2=111.418, df= 162, p= .0001, \chi^2/df= 1.797, GFI= .94, TLI= .94, CFI = .95, RMSEA= .054 [90\% CI= .038 - .070], SRMR= .047)\end{equation*} (Figure 1). Observing correlations of \begin{equation*}r= .19\end{equation*} between PT and CC, \begin{equation*}r=-.26\end{equation*} between PT and WIPS, and \begin{equation*}r= .44\end{equation*} between CC and WIPS. The observed psychometric results methodologically support, as a prior condition, the possibility of estimating the levels of empathy and its dimensions and, therefore, these results contribute to achieving the objective of this study.

### Reliability

The reliability estimated by McDonald’s omega is 0.82, showing adequate internal consistency, with a standardized Cronbach’s alpha of 0.79.

### Associations

The results of the association of the variable age and empathy (in its dimensions) were not significant. It was found that age explains very little the behavior of empathy \begin{equation*}(S=0.0622914; R^2\ unadjusted =7.6\%; R^2\ adjusted = 6.6\%)\end{equation*} and in each of its dimensions: Compassionate Care \begin{equation*}(S=0.0622914; R^2\ unadjusted =7.8\%; R^2\ adjusted= 6.8\%)\end{equation*} , Perspective Talking \begin{equation*}(S=0.06418; R^2\ unadjusted =1.1; R^2 adjusted =0.7\%)\end{equation*} and Walking in Patient’s Shoes \begin{equation*}(S=0.191302; R^2\ unadjusted=0.7\%; R^2\ adjusted=0.0\%)\end{equation*} .

### Descriptive analysis

The results of the estimation of the descriptive statistics are presented in Table 1. The highest CV values are concentrated in the CC and WIPS dimensions, revealing the heterogeneity of the data, unlike the PT dimension and the total empathy score which show homogeneity.

**Table 1 -:** Descriptive statistics of empathy and its dimensions according to types of shifts in which practicing nurses work. Cuenca Province, Ecuador, 2022

	n	M^†^	SD^‡^	CV(%)^§^	95% CI* for the mean		Min.	Max.
					Lower limit	Upper limit		
Empathy								
Full time	252	70.52	12.50	14.8	68.97	72.02	42	95
Half day or less	19	71.53	7.78	10.4	67.78	75.27	53	84
Total	271	70.59	12.22	14.5	67.78	75.27	42	95
Compassionate Care								
Full time	252	29.65	9.83	30.7	28.43	30.87	6	42
Half day or less	19	32.00	6.16	19.2	29.03	34.97	15	41
Total	271	29.81	9.62	30.0	28.43	34.97	6	42
Perspective Taking								
Full time	252	31.42	4.19	12.9	30.90	31.94	7	35
Half day or less	19	31.26	3.53	11.6	29.56	32.96	22	35
Total	271	31.41	4.14	12.8	29.56	32.96	7	35
Walking in Patient Shoes								
Full time	252	6.26	2.858	45.7	5.91	6.62	2	14
Half day or less	19	6.05	2.934	48.5	4.64	7.47	2	12
Total	271	6.25	2.859	45.7	5.91	6.59	2	14

*CI = Confidence interval; ^†^M = Arithmetic average; ^‡^SD = Standard deviation; ^§^CV(%) = Coefficient of variation

Table 2 shows the results of the comparison of empathy and its dimensions between the types of working schedules. The test was not significant (p≥0.05) in all cases, which implies that there are differences between the means compared, assuming eminently equal means.

**Table 2 -:** Comparison of empathy means and their dimensions according to the type of work shift of nurses. Cuenca Province, Ecuador, 2022

	Full time		Half day or less			
Variable	M^*^	SD^†^	M^*^	SD^†^	Z^‡^	p^§^
Empathy	70.52	12.50	71.53	7.78	-0.020	0.984
Compassionate Care	29.65	9.83	32.00	6.16	-0.599	0.549
Perspective Taking	31.42	4.19	31.26	3.53	-0.736	0.461
Walking in Patient´s Shoes	9.46	3.56	8.26	3.02	-1.618	0.106

*M = Arithmetic average; ^†^SD = Standard deviation; ^‡^Z= U of Mann-Whitney; ^§^p= p ≥ 0.05 is not significant

## Discussion

Empathy is a complex construct. Its roots are found in the phylogenetic development and the ontogeny of the subject of the human species^(^ 43 ^-^ 44 ^)^. The phylogenetic component is still active and its action could be expressed through a “synthesis of development systems where morphological inheritance, motor skills, and socio-ecological factors converge”^(^ 36 ^)^, but this development is characterized by the fact that the mechanisms that install quantitative and, above all, qualitative changes are extremely slow^(^ 44 ^-^ 45 ^)^. It is then inferred that the development of empathy in a person is fundamentally modulated by the influence of the processes associated with ontogeny^(^ 46 ^-^ 47 ^)^.

Empathy has cognitive and affective components or dimensions^(^ 48 ^)^. These components interact with each other dialectically. The interactions between the dimensions of empathy materialize in neural networks and the properties of these networks are essentially referred to as a flow of information between them. The interactions between networks may be different (different flows), which could also determine different functional organizations of the network and, therefore, may give rise to different traits of empathy^(^ 48 ^-^ 50 ^)^.

The exact cause of this difference is unknown, but it could relate to topological networks with unequal characteristics that determine individual differences in the dimensions of empathy^(^ 49 ^)^. The formation of the specific topology of the networks in each human being will be strongly influenced by external stimulations more than by genotypic potentiality^(^ 45 ^)^. Some of these stimuli can be as specific as the family environment^(^ 51 ^)^ or as general as society as a whole^(^ 50 ^)^. Questions arise from the ideas expressed above. One of them is whether empathy can be developed indefinitely during the lifetime of a human being. It has been suggested that the neurogenesis present in adults shows the possibility of generating brain plasticity and some studies show a high structural and synaptic plasticity in adults^(^ 52 ^)^. However, this plasticity tends to decrease over time^(^ 53 ^)^. From the above, it is inferred that empathy is not an attribute that develops indefinitely and constantly over time^(^ 44 ^,^ 46 ^)^, at least, there are no studies that demonstrate, directly or indirectly, that brain plasticity can contribute significantly to the development of empathy in the course of a person’s life. In fact, it is known that the prefrontal cortex of the brain is responsible for executive functions and, therefore, for the control of cognition^(^ 54 ^)^. This process is reached between the ages of 25 and 30 with the complete maturity of the prefrontal cortex^(^ 55 ^)^. Therefore, the topology described above reaches its definitive structure in the interval of years mentioned. However, this does not mean that a person above this age stops their learning activity associated with the cognitive dimension of empathy.

The issue is that empathy is a system constituted by a close relationship between the cognitive and the emotional processes and the interaction between all the dimensions of empathy and not an attribute resulting from additive properties. Indeed, the finding that increasing age does not imply an increase in empathy (and its dimensions) in the professional nurses examined could be explained, in part, because the networks associated with the development of neural connections within each dimension, as well as between the networks of the different dimensions that constitute empathy (as a system), reached its maturity, and acquired a certain architecture. The differences between the values of empathy (and its dimensions) between “low” and “high”, including the variations observed in these measurements (homogeneous and heterogeneous CV), could be explained by the differentiated grouping of the levels of empathy (and its dimensions), the differentiated individual interactions determined by the empathic architecture achieved in each nursing professional and by the effects of physical and emotional exhaustion^(^ 56 ^)^, all of which would suppose a different empathic response and behavior with the patient.

The absence of differences between the levels of empathy and the dimensions observed between the type of working schedules of the professional nurses could be explained by the same arguments already raised as possible explanations for the absence of association between age and empathy. The possible empathic response is already determined by the topological neural architecture achieved. Therefore, professional nurses would not have to modify their empathic attitude towards the patient due to work pressure, but rather the mentioned attitude could not exceed the threshold that the topological architecture reached would allow.

The essential basis of the possible explanations of the empirical findings lies in the fact that the development of the cognitive structures of the brain (associated with the cognitive dimensions of empathy) can continue to develop with age, but with the affective dimension, the situation is different. The emotional formation is strongly influenced by ontogeny factors that operate from the earliest childhood^(^ 57 ^-^ 58 ^)^. Child abuse, for example, seriously alters neurological development and delays brain maturation. The consequences can fluctuate from the affectation of attention capacity to the deficit of intellectual development. It negatively influences the processes of neurogenesis, myelination, and neuronal pruning, with consequences on the limbic system and the cerebral cortex. The brain vulnerability hypothesis shows us that “damage may imply a subsequent neurodevelopment that will not be equivalent to the path it could take without the damage produced”^(^ 32 ^)^ and that brain plasticity, in adults, would not be sufficiently effective to repair this damage. If the damage finally occurs, it will affect (to one degree or another) the neurobiological conformation of the components of the limbic system and with this, the ability to generate the necessary interconnections of the network associated with the affective dimension (feeling of compassion) will also be affected. Depending on the degree of affectation, the network that emerges from the affected dimension will not be able to interact adequately with the rest of the networks generated for the other (cognitive) dimensions. Of course, in normal subjects, there are no damages as severe as those described, but there are non-severe “damages” that affect the development of empathy as a whole.

The variability of the levels of empathy observed in the sample of nursing professionals in this study (with almost extreme maximum and minimum values) raises the urgent need to take measures in relation to the empathic behavior of the nurses studied. The causes that originate these results, in light of the theoretical elements exposed, could be explained by the presence of problems that have not yet been solved in the empathic training of nursing students and health sciences in general^(^ 59 ^)^. It is known that the humanization process in patient care is multifactorial and the empathy of the nursing staff with the patient is an important element of it. But if it is affirmed that humanization must be built from patient care training^(^ 60 ^)^, empathy with the patient in the nursing profession must also be built longitudinally and from the first year of training with nursing professionals^(^ 7 ^,^ 20 ^,^ 25 ^-^ 26 ^)^.

The possible contribution of this work could be summarized in the following points: a) The scarcity of studies that evaluate these levels in practicing nursing professionals should be a matter of concern for the corresponding researchers; b) The presence of relatively low levels of empathy in practicing nursing professionals is a finding that must be studied and determine the possible causes that produce it; c) The absence of association between age and empathy is a frequent finding in studies of empathy with the patient. This finding, however, has not been associated with ontogenetic processes imbricated in empathy training, among them, the training process during their stage as a nursing student and d) The absence of differences in the levels of empathy between nursing professionals with different workloads is a finding that should be studied and exceeds the objectives of this study.

The limitations of this study can be systematized in the following points: a) The sample size is not representative of the population studied. For ethical reasons, studies of this type are characterized by the fact that participation is voluntary, and their results are rarely extrapolated to the population studied; b) It was only performed in a hospital in a region of Ecuador. Therefore, its results cannot be extrapolated to the population of nursing professionals working in other hospitals in this country and c) The comparisons between the subsamples of nursing professionals, with different workloads, were distributed with different sample sizes. Taking these limitations into account, the results and findings found should be considered only as trends. It is recommended to continue these studies in different hospitals in Ecuador, as well as in the rest of Latin America.

## Conclusion

In the studied sample of professional nurses, the levels of empathy are relatively low and are not associated with age or the type of work schedule. The values observed in the levels of empathy (especially in the CC and WIPS dimensions) in some of the nursing professionals studied could imply poor performance in empathic patient care.

The main limitation of this work lies in the fact that the results observed were obtained from a non-random sample (due to the characteristics of the work) and with a sample that does not exceed 50% of the population, all of which can lead to concluding terms of trends about the population of nurses examined.
